# Diabetic Ketoacidosis and Profound Insulin Resistance From Brentuximab Vedotin

**DOI:** 10.7759/cureus.35804

**Published:** 2023-03-05

**Authors:** Keval Thakkar, Sonali Khurana, Yujiao Sun, Timothy N Hembree

**Affiliations:** 1 Internal and Hospital Medicine, Moffitt Cancer Center, Tampa, USA; 2 Pharmacy, Moffitt Cancer Center, Tampa, USA

**Keywords:** hyperglycemia, insulin resistance, diabetic ketoacidosis, hodgkin’s lymphoma, adverse drug reaction

## Abstract

Hodgkin’s lymphoma is commonly treated with a combination of chemotherapy drugs including doxorubicin, bleomycin, vinblastine, and dacarbazine. Antibody-drug conjugates such as brentuximab vedotin are now being used to treat Hodgkin’s lymphoma that has not responded to standard treatment. Brentuximab vedotin is a monoclonal antibody that selectively delivers a cytotoxic agent, monomethyl auristatin E, which targets cells expressing surface CD30 markers, a protein that may be found in high amounts in some cancer cells including lymphoma cells. Common adverse effects of the drug include diarrhea, nausea, anemia, and fatigue. We present a case of a patient with diabetic ketoacidosis and profound insulin resistance secondary to brentuximab. Diabetic ketoacidosis is a rare but serious adverse reaction in this growing class of antibody-drug conjugates.

## Introduction

Brentuximab vedotin is an antibody-drug conjugate that targets surface antigens present on tumor cells and releases highly potent anti-cancer agents linked via a chemical linker [1]. It is currently FDA-approved for refractory or relapsed Hodgkin's lymphoma (HL) or relapsed systemic anaplastic large-cell lymphoma [2]. Common toxicities attributed to brentuximab include nerve damage, fatigue, nausea, diarrhea, anemia, and leukocytopenia [3]. Some rare adverse effects, including progressive multifocal leukoencephalopathy, toxic epidermal necrolysis, and severe insulin resistance, have also been reported with it and are thought to be related to immunomodulation or severe inflammation [4]. A few publications have reported diabetic ketoacidosis (DKA), a potentially lethal condition, as one of these potential side effects. Early diagnosis and treatment of DKA can help prevent treatment-related mortality. Here, we present a case of a patient who developed DKA and profound insulin resistance secondary to brentuximab. Given the swift diagnosis, the patient’s DKA was reversed with no lingering insulin resistance or hyperglycemia.

## Case presentation

A 47-year-old female with relapsed HL was referred to an urgent care center by her hematologist because of hyperglycemia, with a blood glucose level of 573 mg/dL. The patient reported progressively worsening nausea, generalized body pain, fatigue, lightheadedness, and decreased appetite. The patient had begun receiving brentuximab vedotin two weeks prior to this presentation and had thus far received one dose of 1.8024 mg/kg (135 mg). She had no prior history of diabetes, and her most recent glycated hemoglobin (HbA1c) percentage from about three months prior was 4.5%.

On physical examination, her weight was 72.5 kg, her height was 164 cm, and she had a body mass index 26.96 kg/m^2^. Her vital signs were normal aside from sinus tachycardia, with a heart rate of 104 beats/minute. In addition to hyperglycemia, laboratory tests revealed an anion gap of 12 mEq/L and positive ketones on urinalysis (Table 1). The patient was treated with 12 units of regular insulin, 40 mEq of intravenous potassium chloride, and 1 L of normal saline (NS). About four hours later, the patient's serum glucose was 399 mg/dL and her symptoms had resolved. She was prescribed 500 mg of metformin daily and 100 mg of sitagliptin daily, then discharged home.

**Table 1:** Patient’s laboratory test results on first and second visits pCO2: partial pressure of carbon dioxide; HCO3: bicarbonate

Analysis	Value at first visit	Value at second visit	Normal range
Serum chemistry panel			
Sodium, mmol/L	131	133	135-145
Potassium, mmol/L	3.8	3.2	3.3-5.0
Chloride, mmol/L	97	98	98-107
Blood glucose, mg/dL	573	430	65-110
Total bicarbonate, mmol/L	22	17	21-32
Hemoglobin A1C, (%)	–	6.7	4.8-5.9
Serum acetone/ketone, mg/dL	–	13	<5
Anion gap, mmol/L	12	18	4-12
Arterial blood gas			
pH	–	7.321	7.35-7.45
pCO2, mmHg	–	31.3	34-35
HCO3, mEq/L	–	16.1	22-26
Urinalysis			
Glucose, mg/dL	50	100	None
Ketone, mmol/L	40	≥160	None

Four days later, the patient returned to the hematology clinic and was referred again to the urgent care center for hyperglycemia. Laboratory workup revealed a blood glucose level of 430 mg/dL, an anion gap of 28 mmol/L, and a hemoglobin A1c of 6.7% (Table 1). The patient was alert and oriented, endorsed nausea without vomiting, and denied experiencing any chest pain, shortness of breath, diarrhea, or abdominal pain. Apart from tachycardia, with a heart rate of 119 beats/min, her vital signs remained stable. She was prescribed 1 L of NS and 18 units of regular insulin and was admitted to the intensive care unit with a diagnosis of DKA.

The patient received an insulin infusion of 0.1 units/kg/hour, an NS infusion, and potassium replacement. Despite her fasting insulin requirement reaching above the institution's maximum hourly rate of 0.6207 units/kg/hour (1080 units per 24 hours), her DKA persisted for roughly four days. On her fourth day of admission, the patient was prescribed a low carbohydrate diet as well as 10 units of insulin glargine and four units of insulin lispro three times daily with meals, in addition to her preexisting insulin infusion requirement. On her fifth day of admission, she received 50 units of insulin glargine in the morning and evening, and on her sixth day, she was weaned off insulin infusion. Her blood glucose remained at the goal of <180 mg/dL on the seventh day, with only two units of additional insulin lispro received throughout the day. Bedtime insulin glargine was discontinued on the seventh day of admission, and prandial insulin lispro was discontinued on her eighth day. The patient was discharged to home on the 10th day of hospitalization with no antihyperglycemic medications or insulin therapy. At her follow-up appointment two weeks after hospital discharge, her blood glucose remained normal at 102 mg/dL, and the decision was made to discontinue treatment with brentuximab vedotin.

## Discussion

Though widespread use of targeted immunotherapies, including brentuximab vedotin, have altered the treatment landscape for HL, they can also cause a wide range of drug toxicities. In August 2019, the FDA Adverse Events database reported 70 cases of hyperglycemia related to brentuximab vedotin, 25 reports of DKA, and 12 cases of cytokine release syndrome [5]. To our knowledge, this is the first case reported in the state of Florida of extreme insulin resistance and DKA after brentuximab infusion alone for a patient with no prior history of diabetes, hyperglycemia, or other high-risk medical conditions.

In similar cases, it is thought that brentuximab contributes to severe cytokine release and thus extreme insulin resistance. One case study suggested that severe insulin resistance and DKA in the context of hyperinsulinemia was likely due to impaired insulin signaling induced by the combination of brentuximab and cyclosporin, rather than brentuximab vedotin alone, for a patient who was treated for relapsed HL [6]. Another case reported severe cytokine release syndrome with brentuximab treatment resulting in vasopressor-dependent shock, oliguria, and increased inflammatory cytokines, leading to multi-organ failure and the patient’s death [7]. Unlike these cases, for our patient, drug-induced hyperglycemia was moderate and reversible, and it was thought to be directly related to brentuximab alone rather than the contribution of other drug therapies.

The CD30 protein, which is targeted by brentuximab vedotin, is part of the tumor necrosis factor receptor superfamily expressed in regulatory T cells, and it plays a protective role in autoimmune diseases [8]. Depletion of CD30 leads to compromised negative selection in the thymus and increased T-cell autoreactivity blockade of the CD30 pathway. This reduces protection by regulatory T cells from pro-inflammatory cytokine accumulation and T-cell apoptosis [9]. These pro-inflammatory cytokines can cause insulin resistance in adipose tissue, skeletal muscle, and the liver by inhibiting insulin signal transduction, therefore causing hyperglycemia and possible DKA [10].

DKA caused by brentuximab is extremely rare yet life-threatening. Thus, increased awareness of the hyperglycemic side effects of brentuximab is crucial and should be identified early to ensure the prevention of DKA and reduction of mortality. This case highlights the positive clinical outcomes made possible by swift clinical action to combat brentuximab-induced hyperglycemia and demonstrates the importance of monitoring for hyperglycemic side effects when prescribing this drug class.

## Conclusions

DKA due to brentuximab treatment for HL is a rare condition. We suspect that brentuximab administration can lead to systemic cytokine release with extreme insulin resistance, as evidenced by this case. With increased usage of targeted immunotherapies in cancer patients, treating physicians must be aware and vigilant for possible inflammatory and autoimmune adverse effects caused by brentuximab and other antibody-drug conjugates. Additional studies are needed to elucidate specific patient risk factors that may increase susceptibility to rare inflammatory or immune-related events from targeted immunotherapies.
